# Supplementary material for: Metabolic, inflammatory and adipokine differences on overweight/obese children with and without metabolic syndrome: A cross-sectional study
Source: PLoS One. 2023 Mar 15;18(3):e0281381. doi: 10.1371/journal.pone.0281381 (PMC10016645; doi:10.1371/journal.pone.0281381)
Supplement: S1 Checklist — (DOCX) [file pone.0281381.s001.docx]

STROBE Statement—checklist of items that should be included in reports of observational studies

|  | Item No. | Recommendation | Page  No. | Relevant text from manuscript |
| --- | --- | --- | --- | --- |
| **Title and abstract** | 1 | (*a*) Indicate the study’s design with a commonly used term in the title or the abstract | 1 | This was an observational study. |
|  |  | (*b*) Provide in the abstract an informative and balanced summary of what was done and what was found | 1 | Abstract with methods and results |
| Introduction | | | |  |
| Background/rationale | 2 | Explain the scientific background and rationale for the investigation being reported | 2-3 | Obesity is also linked to changes in serum lipoproteins […] Obesity is related to both classic and novel risk factors, including prothrombotic factors, inflammatory factors, and some adipocytokines. |
| Objectives | 3 | State specific objectives, including any prespecified hypotheses | 4 | The objective herein was to explore the profile of adipokines, inflammatory markers, and lipid ratios to identify children with cardiometabolic risk. |
| Methods | | | |  |
| Study design | 4 | Present key elements of study design early in the paper | 4-6 | We described these sections: Sample selection, definitions, data collection, statistical analysis. |
| Setting | 5 | Describe the setting, locations, and relevant dates, including periods of recruitment, exposure, follow-up, and data collection | 4 | This was an observational study of children and adolescents attending pediatric consultation at the University Hospital “Dr. José Eleuterio González” of the Autonomous University of Nuevo León in Monterrey, N.L., Mexico conducted between January 2017 and December 2019. The institutional ethics committee approved the study (PE17-00010). A detailed letter explaining the study aims was provided to all parents or guardians and informed consent was obtained |
| Participants | 6 | (*a*) *Cohort study*—Give the eligibility criteria, and the sources and methods of selection of participants. Describe methods of follow-up  *Case-control study*—Give the eligibility criteria, and the sources and methods of case ascertainment and control selection. Give the rationale for the choice of cases and controls  *Cross-sectional study*—Give the eligibility criteria, and the sources and methods of selection of participants | 4 | The inclusion criteria were: (I) younger than age 18 years; and (II) body mass index (BMI) ≥85th percentile according to the Centers for Disease Control and Prevention (CDC). The exclusion criteria were: (I) congenital malformation; (II) previous diagnosis with endocrinological, kidney, or hepatic disorder; (III) use of any medication affecting serum lipid concentration; and (IV) refusal to participate in the study. |
|  |  |  |  |  |
| Variables | 7 | Clearly define all outcomes, exposures, predictors, potential confounders, and effect modifiers. Give diagnostic criteria, if applicable | 5 | Overweight and obesity were defined according to the criteria established by the CDC. Overweight was considered a BMI between the 85th and 95th percentiles. Obesity was considered a BMI ≥95th percentile.  MetS was defined according to the de Ferranti criteria […] |
| Data sources/ measurement | 8* | For each variable of interest, give sources of data and details of methods of assessment (measurement). Describe comparability of assessment methods if there is more than one group | *5-6* | In the data collection section, we described each variable of interest. |
| Bias | 9 | Describe any efforts to address potential sources of bias | *5-6* | In the data collection section, we described each variable of interest. |
| Study size | 10 | Explain how the study size was arrived at | 4 | […] of children and adolescents attending pediatric consultation at the University Hospital “Dr. José Eleuterio González” of the Autonomous University of Nuevo León in Monterrey, N.L., Mexico conducted between January 2017 and December 2019. |

Continued on next page

| Quantitative variables | 11 | Explain how quantitative variables were handled in the analyses. If applicable, describe which groupings were chosen and why | 6 | Normally distributed variables are presents as means and standard deviations and were analyzed by Student´s t tests. Non-normally distributed variables […] Reciever operating characteristic (ROC) analysis was performed to determine the area under the curve […] |
| --- | --- | --- | --- | --- |
| Statistical methods | 12 | (*a*) Describe all statistical methods, including those used to control for confounding | 6 | Student´s t-test, Mann-Whitney tests. ROC analysis, Spearman correlation. |
|  |  | (*b*) Describe any methods used to examine subgroups and interactions | NA |  |
|  |  | (*c*) Explain how missing data were addressed | NA |  |
|  |  | (*d*) *Cohort study*—If applicable, explain how loss to follow-up was addressed  *Case-control study*—If applicable, explain how matching of cases and controls was addressed  *Cross-sectional study*—If applicable, describe analytical methods taking account of sampling strategy | NA |  |
| Results | | | | |
| Participants | 13* | (a) Report numbers of individuals at each stage of study—eg numbers potentially eligible, examined for eligibility, confirmed eligible, included in the study, completing follow-up, and analysed | 7 | The total sample was 107 patients, among whom 63 were male (58.8%) and 44 were female (41.1%); their mean age was 10.52±1.76 years. Among the total sample, 21 children (19.6%) had normal body weight and 86 (80.4%) had overweight/obesity. |
|  |  | (b) Give reasons for non-participation at each stage | NA |  |
|  |  | (c) Consider use of a flow diagram | NA |  |
| Descriptive data | 14* | (a) Give characteristics of study participants (eg demographic, clinical, social) and information on exposures and potential confounders | 8 | Table 1. Anthropometric and laboratory parameters of the children |
|  |  | (b) Indicate number of participants with missing data for each variable of interest | NA |  |
|  |  | (c) *Cohort study*—Summarise follow-up time (eg, average and total amount) | NA |  |
| Outcome data | 15* | *Cohort study*—Report numbers of outcome events or summary measures over time | *NA* |  |
|  |  | *Case-control study—*Report numbers in each exposure category, or summary measures of exposure | *7* | The total sample was 107 patients, among whom 63 were male (58.8%) and 44 were female (41.1%); their mean age was 10.52±1.76 years. Among the total sample, 21 children (19.6%) had normal body weight and 86 (80.4%) had overweight/obesity. |
|  |  | *Cross-sectional study—*Report numbers of outcome events or summary measures | *NA* |  |
| Main results | 16 | (*a*) Give unadjusted estimates and, if applicable, confounder-adjusted estimates and their precision (eg, 95% confidence interval). Make clear which confounders were adjusted for and why they were included | NA |  |
|  |  | (*b*) Report category boundaries when continuous variables were categorized | NA |  |
|  |  | (*c*) If relevant, consider translating estimates of relative risk into absolute risk for a meaningful time period | NA |  |

Continued on next page

| Discussion | | | | |
| --- | --- | --- | --- | --- |
| Key results | 18 | Summarise key results with reference to study objectives | 17 | In sum, the main risk factors associated with metabolic disease and cardiovascular risk were WC, hypertension, atherogenic dyslipidemia (elevated TC, low HDL-C), and inflammatory parameters (CRP, PAI-1). Of note, both TG/HDL and TC/HDL-C are available from routine lab tests, simplifying surveillance for cardiovascular risk among children with overweight or obesity. |
| Limitations | 19 | Discuss limitations of the study, taking into account sources of potential bias or imprecision. Discuss both direction and magnitude of any potential bias | 17 | To date, few studies in Mexico have evaluated the cardiovascular risk indices in children. Therefore, some limitations of the present study must be acknowledged. |
| Interpretation | 20 | Give a cautious overall interpretation of results considering objectives, limitations, multiplicity of analyses, results from similar studies, and other relevant evidence | 17 | In sum, the main risk factors associated with metabolic disease and cardiovascular risk were WC, hypertension, atherogenic dyslipidemia (elevated TC, low HDL-C), and inflammatory parameters (CRP, PAI-1). Of note, both TG/HDL and TC/HDL-C are available from routine lab tests, simplifying surveillance for cardiovascular risk among children with overweight or obesity. |
| Generalisability | 21 | Discuss the generalisability (external validity) of the study results | NA |  |
| Other information | |  | | |
| Funding | 22 | Give the source of funding and the role of the funders for the present study and, if applicable, for the original study on which the present article is based | 18 | The authors did not receive support from any organization for the submitted work. |

*Give information separately for cases and controls in case-control studies and, if applicable, for exposed and unexposed groups in cohort and cross-sectional studies.

**Note:** An Explanation and Elaboration article discusses each checklist item and gives methodological background and published examples of transparent reporting. The STROBE checklist is best used in conjunction with this article (freely available on the Web sites of PLoS Medicine at http://www.plosmedicine.org/, Annals of Internal Medicine at http://www.annals.org/, and Epidemiology at http://www.epidem.com/). Information on the STROBE Initiative is available at www.strobe-statement.org.
