# Supplementary material for: Metabolic, inflammatory and adipokine differences on overweight/obese children with and without metabolic syndrome: A cross-sectional study
Source: PLoS One. 2023 Mar 15;18(3):e0281381. doi: 10.1371/journal.pone.0281381 (PMC10016645; doi:10.1371/journal.pone.0281381)
Supplement: Attachment — Submitted filename: Response to Reviewers.docx [file pone.0281381.s005.docx]

Dear Editors,

We would like to thank the reviewers for their valuable observations about our manuscript entitled “Metabolic, inflammatory and adipokine differences on overweight/obese children with and without metabolic syndrome: a cross-sectional study”. We have edited the manuscript according to the modifications kindly requested by the reviewers.

We believe that our manuscript is now suitable for its publication in PlosOne.

Best of my regards.

Paula Cordero Pérez, PhD.

Liver Unit, Department of Internal Medicine.

University Hospital “Dr. José E. González”

Universidad Autónoma de Nuevo León.

On behalf of all authors.

Reviewer 2

**• Please, move the p-value at the end of each sentence. Eg. “(p=0.002, OR=0.88, 95% CI=0.81-0.95)” should be “(OR=0.88, 95% CI=0.81-0.95, p=0.002)”**

*We appreciate the observation, and this has already been corrected in the lineS 180 to 182*

**• In the Adipo/Lep ratio 6.68(7.89), what does the number in parenthesis represents? If it’s the standard deviations, please clarify.**
*We appreciate the observation, and this has been clarify in the Table 1.*

**• In table 3, please round to three decimals the correlation coefficient and the p-value. Additionally, please include the 95% confidence intervals within both types of correlations.**

*The expression of the decimals has already been unified, and 95% confidence intervals have been added.*

**• In Page 19 and Line 349-350: The label “Fig 1. Frequency of the Ferranti criteria used for the diagnosis of metabolic syndrome.” seems out of line.**

*Figure captions were inserted immediately after the first paragraph in which the figure was cited, and these captions were aligned to the text.*
 **• In figure 1, please specify in the x-axis that this is the prevalence.**

*We appreciate the observation, and this has already been corrected*
